# Effects of HPV-related psychosocial burden and general psychological health on quality of life and sexual function in women with HPV infection in the initial period after diagnosis

**DOI:** 10.1192/bjo.2025.10943

**Published:** 2026-02-02

**Authors:** Sofia Boukouvala, Themistoklis Loukopoulos, Antonios Athanasiou, Maria Kyrgiou, Minas Paschopoulos, Evangelos Paraskevaidis, Vassiliki Siafaka

**Affiliations:** University Hospital of Ioannina, Ioannina, Greece; Department of Obstetrics & Gynaecology, University Hospital of Ioannina, Ioannina, Greece; Department of Metabolism, Digestion and Reproduction, Institute of Reproductive and Developmental Biology, Imperial College London, London, UK; Department of Obstetrics & Gynaecology, Faculty of Medicine, School of Health Sciences, University of Ioannina, Ioannina, Greece; School of Health Sciences, University of Ioannina, Ioannina, Greece

**Keywords:** Women with HPV, quality of life, psychological distress, sexual function, initial period

## Abstract

**Background:**

Human papillomavirus (HPV) infection has a negative impact on quality of life (QoL) and sexual function, mainly owing to increased levels of anxiety and distress.

**Aims:**

To examine the potentially moderating effects of general psychological health on the relationships between (a) HPV-related psychosocial burden and QoL and (b) HPV-related psychosocial burden and sexual function.

**Method:**

The HPV Impact Profile, Female Sexual Function Index, General Health Questionnaire-28 and Life Satisfaction Inventory questionnaires were completed by 151 women.

**Results:**

HPV-related psychosocial burden and general psychological health accounted for 23.2% of QoL variability. There was not strong evidence for a moderating effect of general psychological health on the relationship between HPV-related psychosocial burden and QoL. Higher HPV-related psychosocial burden predicted worse sexual function on average. However, HPV-related psychosocial burden accounted for only 4.1% of sexual function variability.

**Conclusions:**

Higher HPV-related psychosocial burden is associated with lower QoL as well as worse sexual function. General psychological health predicts changes in QoL over and above HPV-related psychosocial burden; thus, a deep understanding of emerging mental health issues soon after diagnosis is crucial to improve counselling and enhance women’s mental empowerment to achieve a better psychological response.

Human papillomavirus (HPV) is among the most common sexually transmitted infections worldwide according to data from the Centers for Disease Control and Prevention.^1^ The infection mainly affects adolescents and young individuals after they have become sexually active.^1,2^ It is estimated that in developed countries, 1–2% of sexually active women aged 16–45 years will be infected, 4% will have a subclinical form of the disease and 15% will be healthy carriers. The maximum susceptibility to infection is estimated to occur between the ages of 16–25 years.^3^

Diagnosis of HPV is expected to have an impact on patients’ emotional state and, by extension, on their psychosocial health and sexuality. The infection mainly affects the vulva and the cervix in women; therefore, it is not uncommon for patients to develop feelings of shame or reduced femininity, and for this reason HPV infection can affect the patient’s sexuality and her relationship with her partner.^4,5^ In addition, potential malignant mutation of the lesions may evoke fear and anxiety.^6–8^ Repeated examinations and contact with doctors, as well as the invasive nature of treatment, can also have a negative effect on sexuality. Previous studies have demonstrated that HPV infection has a negative effect on both psychological and sexual health of patients.^9,10^ In addition, it has been well established that HPV infection has a negative impact on quality of life (QoL), mainly owing to increased levels of anxiety and distress.^11–14^ However, given that decreased QoL may be related to both HPV-related and non-HPV-related sources, to assess the unique effect of HPV-related psychosocial burden on QoL it is important to also account for the role of the more generic psychological health with respect to QoL. We hypothesised that after accounting for differences in general psychological health, there would still be a substantial effect of HPV-related psychological burden on QoL and sexual function. Therefore, the purpose of the present study was to assess the moderating effects of general psychological health on the relationships between (a) HPV-related psychosocial burden and QoL and (b) HPV-related psychosocial burden and sexual function.

## Method

### Participants

The study took place at the out-patient cervical pathology and colposcopy clinic of a tertiary university hospital. All procedures contributing to this work comply with the ethical standards of the relevant national and institutional committees on human experimentation and with the Helsinki Declaration of 1975, as revised in 2013. In addition, the study protocol was approved by the local ethics research committee (no. 839/26.10.2021). A written statement of agreement to participate in this study was provided by each participant before entering the study. On the basis of the international literature, approximately 150 women aged 18–45 years were invited to participate in this study after their first visit to the out-patient cervical pathology and colposcopy clinic. Inclusion criteria for the study included cytological examination suspicious for HPV or for cellular atypia, ability to understand and read the Greek language, absence of history of depression, and absence of organic or functional pathology. Women positive for HPV infection and those with obvious cervical lesions that were scheduled for immediate medical or surgical treatment were also excluded from the present study.

### Procedures

This study followed a cross-sectional design. Women that met the inclusion criteria were identified by their physician and invited by one of the researchers to participate in the study. Participation was voluntary, and data confidentiality was guaranteed. After signing the informed consent form, women answered the questionnaires on the day of their medical appointment in the initial period (1–6 weeks) after the HPV diagnosis.

### Measurement tools

Demographic and clinical data were obtained including age, marital status, educational level, children, number of sexual partners and history of warts. In addition, participants completed the following tools.

- Life Satisfaction Inventory (LSI) to measure QoL. This is a 13-item tool that assesses patients’ satisfaction with physical and mental health, work, financial status, relationship with partner, sexual life, family life, role within family, friends, hobbies, appearance and general QoL during the past week.^15^ The scale shows good internal consistency with Cronbach’s *α* = 0,82. The tool has been translated and validated in a Greek healthy population.^16^
- Female Sexual Function Index to assess sexual function. A version of the original English tool translated into Greek and evaluated for reliability was used.^17^ This tool was developed to assess a woman’s sexual activity in the previous 4 weeks.^18^ It allows assessment, on a scale from 0 (no sexual activity) to 5 (always/very high sexual activity), of sexual desire, arousal, hydration, orgasm, satisfaction and dyspareunia. The score has a range of 2–35, with values ≤26.55 considered to be acceptable for diagnosis of sexual dysfunction among women of a wide age range.^18^ In the Greek version, all six subdomains of the questionnaire demonstrated good internal validity (Cronbach’s *α* = 0.92, *P* < 0.01) and excellent reliability (intraclass correlation coefficient: 0.91, *P* < 0.01).^17^ The authors categorised women with and without sexual dysfunction with a cut-off value of 26 (sensitivity: 71.4%; specificity: 92.2%).
- HPV Impact Profile (HIP) questionnaire to estimate HPV-related psychosocial burden.^19^ This questionnaire contains 29 items that are summarised in seven domains. Answers are given on a 0–10 Likert scale, and total HIP scores range from 0 (no impact) to 100 (worst impact). Its Cronbach’s *α* has been reported to range from 0.64 to 0.90, with values ≥0.7 reported for five of the seven domains. The original version of the HIP tool exhibits acceptable discriminant construct validity and reliability and can be used to estimate the importance of the HPV-related burden.^19^
- General Health Questionnaire-28 to measure general psychological health. This questionnaire is a self-report screening measure used to assess possible psychological disorders and aims to examine inability to carry out normal functions, as well as the appearance of new and distressing phenomena. It comprises four seven-item subscales (somatic symptoms, anxiety/insomnia, social dysfunction and severe depression) and is used to identify the presence of symptoms compared with what is normal for the individual.^20^ A validated Greek version has been published.^21^

### Statistical analysis

For descriptive purposes, observed central tendencies and dispersions are reported using means and standard deviations for continuous variables and proportions or frequencies for categorical data as appropriate.

A Bayesian framework was chosen instead of a frequentist approach for all analyses as this allowed results to be interpreted intuitively through reporting of subjective probabilities.^22^ Briefly, in frequentist linear models, results are interpreted on the basis of *P*-values and confidence intervals. These reflect how likely the observed data are, assuming there is no effect (the null hypothesis). For example, a 95% confidence interval means that if the experiment is repeated many times, 95% of the intervals would contain the true value.^22^ By contrast, Bayesian linear models combine prior knowledge (or assumptions) with the data to directly produce a posterior distribution of the parameter estimates.^22,23^

Consistent with the aim of our analysis, the response variables were QoL and sexual function, with HPV-related psychological burden and general psychological health serving as potential explanatory variables. In addition, baseline demographic and clinical data (age, marital status, educational background, having children, number of sexual partners and history of warts) were used as covariates to control for potential confounding. We tested moderation models both with and without adjustment for baseline covariates to assess whether the inclusion of these variables improved model fit or altered the substantive interpretation of the moderation effect. This approach enabled us to evaluate the robustness of the effect while accounting for potential confounding and/or overfitting.

For the purpose of the moderation analysis, we compared eight competing models: model 1 had HPV-related burden, model 2 had general psychological health, model 3 had both HPV-related burden and general psychological health, and model 4 had HPV-related burden and general psychological health and their interaction as predictors of QoL or sexual function. In addition, models 1–4 were adjusted for age, marital status, children, educational background, number of partners and history of warts. Models 5–8 corresponded to models 1–4 but without adjustment for baseline demographic and clinical data. A substantial interaction (model 4, with covariate adjustment; model 8, without covariate adjustment) would indicate that the effect of HPV-related psychological burden on QoL or sexual function was moderated by general psychological health. In that case, general psychological health would be the moderator of HPV-related psychological burden effect on QoL or sexual function. Identifying a moderator of an effect helps to establish the boundary conditions of that effect or the circumstances, stimuli or type of people for which the effect is large versus small, or present versus absent.^24^ Models were compared and weighted using information criteria based on out-of-sample predictive accuracy. Specifically, we used leave-one-out cross-validation to assess model fit and generalisability. For each model, the expected log predictive density was estimated, and model weights were calculated using the leave-one-out model weighting approach.^24^ This allows probabilistic model comparison and accounts for model uncertainty by estimating how likely each model is to be the best predictor of new data. This approach is particularly suited to Bayesian models, for which conventional likelihood-based criteria (such as the Akaike information criterion or Bayesian information criterion) may not be appropriate or sufficient for comparison of models with different structures or priors. In addition, the Bayesian version of *R* ^2^ was used to quantify the amount of ‘explained’ outcome variance for each model.^23^

The variables used to measure QoL and sexual function (LSI and Female Sexual Function Index, respectively) were log-transformed before analyses; back-transformation of the effects yielded their magnitude in percentage units. In addition, to augment interpretation of the intercept (reference category) and slope (difference between the reference and other categories), HPV-related psychosocial burden (estimated by HIP) was standardised using *Z*-score transformation (thus, ZHIP = 0 refers to the average HIP of the sample). Finally, general psychological health (quantified by the General Health Questionnaire-28) was entered as a categorical predictor (‘no distress’ versus ‘probable distress’) on the basis of a cut-off value ≥5. Inferences from all analyses were performed on posterior samples generated using the Hamiltonian Markov chain Monte Carlo method and through use of 95% credible intervals (CrIs). For all models, inferences were made as one-sided hypotheses, and a posterior probability that exceeded 95% was considered to suggest the existence of evidence for substantial difference.^22^ Weakly informative priors (Student’s *t* and half Student’s *t* with three degrees of freedom for intercepts and variance parameters, respectively) were used in all models; for model effects (slopes or differences between factor levels and reference category), we used weakly informative normal priors (*N* ∼ 0, 10).^22^ Data were analysed using R (version 4.3.2; R Core Team, R Foundation for Statistical Computing, Vienna, Austria) on a Windows 11 platform, employing the brms wrapper package interfaced with the probabilistic programming language Stan for Bayasian sampling (https://www.R-project.org).^25^

## Results

Demographic and clinical characteristics of the participants are provided in Table 1, and Table 2 presents observed values of central tendency and dispersion for all tools.

**Table 1:** Demographic and clinical characteristics of the study population

Variables		*N*	%
Categorical variables			
Age category, years	<30	31	20.5
	30–40	54	35.5
	>40	66	43.7
Marital status	Single	54	35.8
	Married	76	50.3
	Divorced	21	13.9
Children	No	62	41.1
	Yes	89	58.9
Number of sexual partners	<5	74	49.0
	5–10	64	42.4
	>10	13	8.6
History of warts	No	53	35.3
	Yes	97	64.7
General Health Questionnaire-28 (cat)	No distress	100	66.2
	Probable distress	51	33.8
Smoking	No	29	19.3
	Yes	122	80.7
Education	High school	68	45.0
	University	55	36.5
	MSc/PhD	28	18.5
Continuous variables		Mean	s.d.
Age, years		36.9	7.6
Menarche, years		13.1	1.5
Sexual partners, *n*		18	7

**Table 2:** Observed scores (mean ± s.d.) for all measurement tools (*n* = 151)

Tool	Mean	s.d.	Minimum	Maximum
Life Satisfaction Inventory	48.5	8.6	19	65
Female Sexual Function Index	26.6	4.9	15.2	36.0
HPV Impact Profile	31.7	16.2	2.8	78.3
General Health Questionnaire-28	4.5	5.5	0	27

**Fig. 1:**
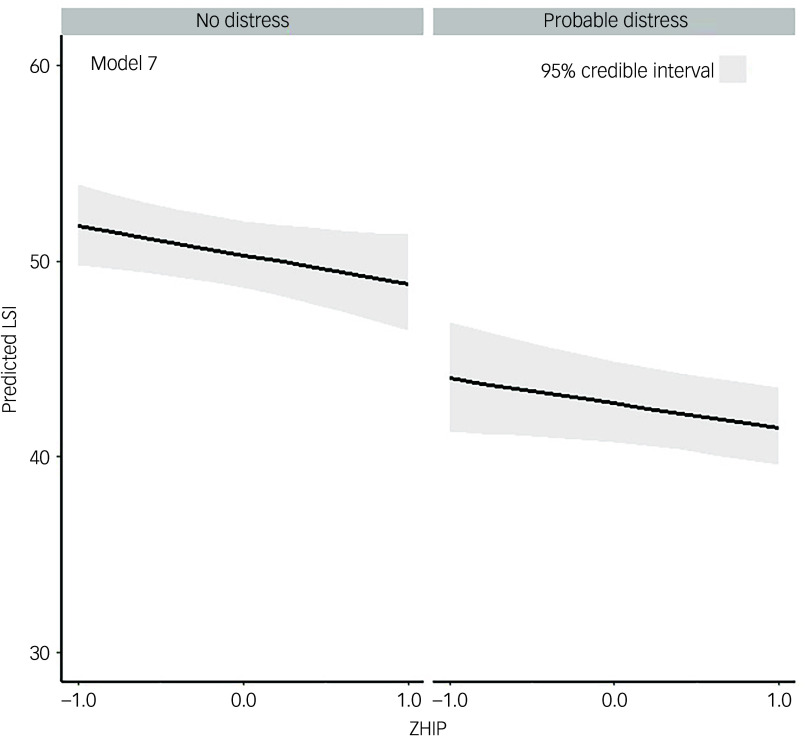
Moderation of general psychological health on the relationship between quality of life and human papillomavirus (HPV)-related psychological burden. Best fitting model; main effects of general psychological health and HPV-related psychological burden without covariate adjustment. LSI, Life Satisfaction Inventory; ZHIP, *Z*-score transformed HPV Impact Profile.

Regarding the moderating effects of general psychological health on the relationship between QoL and HPV-related psychosocial burden, the model with both QoL and HPV-related psychosocial burden as main effects but without adjustment for demographic or clinical covariates (model 7) outperformed all other models (Table 3). Within this model, after general psychological health had been accounted for, higher HPV-related psychosocial burden predicted lower QoL on average (−2.9% [95% CrI: −5.7%; −0.1], 98.0% probability for an effect <0). In addition, after we had accounted for HPV-related psychosocial burden, worse general psychological health predicted lower QoL on average (−15.0% [95% CrI: −20.1%; −9.7%], 99.9% probability for an effect <0) (Fig. 1, model 7). Models without covariate adjustment outperformed their corresponding counterparts with covariate adjustment. In addition, general psychological health alone had a greater impact on QoL compared with HPV-related psychosocial burden alone, given that model 7 accounted for more than twice the amount of outcome variability compared with model 6 (Table 3). In addition, there was no evidence for a substantial moderating effect of general psychological health on the relationship between HPV-related psychosocial burden and QoL (1.5% [95% CrI: −4.3; 7.6], 68.0% probability for an effect >0) (model 4); there was also a trivial increase in the explained outcome variance for the interaction model compared with the simpler model (model 8 versus model 7) (Table 3).

**Table 3:** Comparison of models with respect to effects of human papillomavirus (HPV)-related psychosocial burden and general psychological health on quality of life in women with HPV infection^a^

Parameter	Model 1^b^	Model 2^c^	Model 3^d^	Model 4^e^	Model 5^f^	Model 6^g^	Model 7^h^	Model 8^i^
Intercept	46.8 [43.0, 51.0]	49.0 [45.1, 53.1]	49.4 [45.6, 53.7]	49.4 [45.4, 53.5]	47.6 [46.2, 49.1]	50.7 [48.9, 52.5]	50.3 [48.6, 52.1]	50.2 [48.5, 52.0]
HIP	−5.2% [−8.2, −2.1]	–	−2.4% [−5.5, 0.7]	−2.9% [−6.6, 0.9]	−5.7% [−8.5, −2.9]	–	−2.9% [−5.7, −0.1]	−3.4% [−7.0, 0.2]
GHQ-28	–	−16.0% [−20.8, −11.2]	−14.6% [−19.7, −9.2]	−14.8% [−20.0, −9.3]	–	−16.9% [−21.5, −12.1]	−15.0% [−20.1, −9.7]	−15.3% [−20.3, −10.2]
HIP × GHQ-28	–	–	–	1.3% [−4.7, 7.4]	–	–	–	1.5% [−4.3, 7.6]
Residual variation	20.3% [18.0, 23.1]	18.5% [16.4, 21.0]	18.5 [16.3, 21.0]	18.5 [16.3, 21.1]	20.9% [18.4, 23.7]	19.0% [16.9, 21.5]	18.9 [16.7, 21.4]	18.9 [16.7, 21.5]
Bayesian *R* ^2^	18.8 [10.4, 27.6]	29.1 [19.2, 38.3]	30.6 [20.9, 39.4]	30.7 [21.0, 39.4]	9.3 [2.3, 18.0]	20.7 [10.8, 30.5]	23.0 [12.8, 32.8]	23.5 [13.5, 33.1]
Model weights	0.0	11.2	11.3	4.1	0.0	22.6	37.7	13.1

HIP, HPV Impact Profile; GHQ-28, General Health Questionnaire-28; LSI, Life Satisfaction Inventory; ZHIP, *Z*-score transformed HIP.

a. Estimates are reported as mean [95% credible interval].

b. LSI ∼ 1 + ZHIP + baseline adjustment (age, marital status, children, educational background, number of partners, history of warts). (The ‘~’ acts as a formula separator in each statistical model, meaning ‘is modeled by’ or ‘is a function of’.)

c. LSI ∼ 1 + GHQ-28 + baseline adjustment.

d. LSI ∼ 1 + ZHIP + GHQ-28 + baseline adjustment.

e. LSI ∼ 1 + ZHIP + GHQ-28 + baseline adjustment.

f. LSI ∼ 1 + ZHIP.

g. LSI ∼ 1 + GHQ-28.

h. LSI ∼ 1 + ZHIP + GHQ-28. This model was the final selected model.

i. LSI ∼ 1 + ZHIP + GHQ-28; baseline adjustment.

**Fig. 2:**
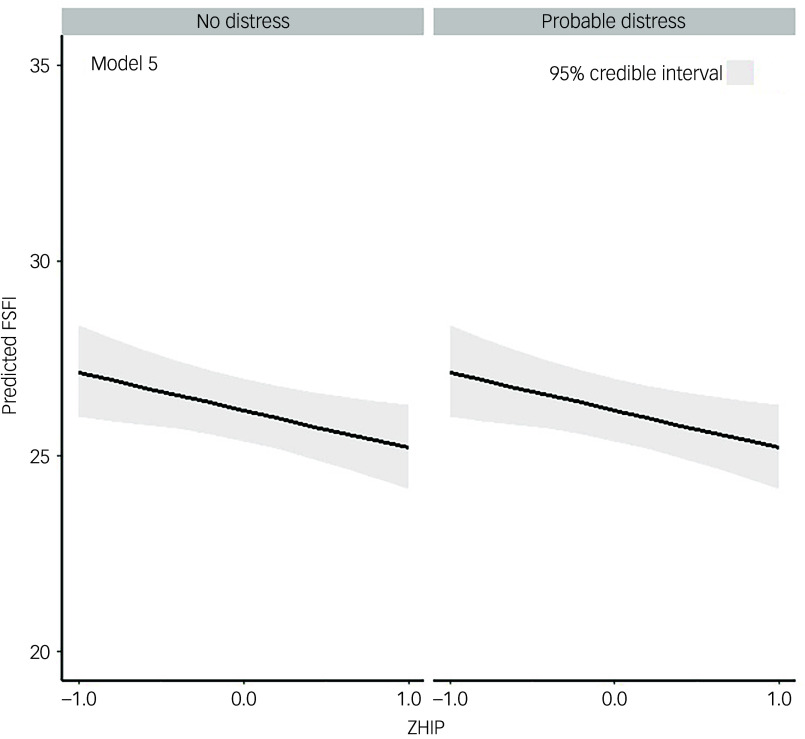
Moderation of general psychological health on the relationship between sexual function and human papillomavirus (HPV)-related psychological burden. Best fitting model; main effect of HPV-related psychological burden without covariate adjustment. FSFI, Female Sexual Function Index; ZHIP, *Z*-score transformed HPV Impact Profile.

Regarding the moderating effects of general psychological health on the relationship between sexual function and HPV-related psychosocial burden, the model with HPV-related psychosocial burden as the single predictor but without covariate adjustment (model 5) outperformed all other models (Table 4). Within this model, there was strong evidence that higher HPV-related psychosocial burden predicted worse sexual function on average (−3.6% [95% CrI: −6.4; −0.7], 99.0% probability for an effect <0) (Fig. 2). As a consequence, there was no strong evidence for a moderating effect of general psychological health on the relationship between HPV psychosocial burden and sexual function (4.0% [95% CrI: −2.3; 10.6], 89.0% probability for an effect >0). Again, models without covariate adjustment outperformed their counterparts with covariate adjustment. Notably, the amount of explained sexual function variance was only ∼4% for the best-performing model (Table 4, model 5).

**Table 4:** Comparison of models with respect to effects of human papillomavirus (HPV)-related psychosocial burden and general psychological health on sexual function in women with HPV infection^a^

Parameter	Model 1^b^	Model 2^c^	Model 3^d^	Model 4^e^	Model 5^f^	Model 6^g^	Model 7^h^	Model 8^i^
Intercept	27.1 [24.8, 29.5]	27.0 [24.6, 29.5]	27.4 [25.0, 29.9]	27.2 [24.9, 29.8]	26.2 [25.4, 27.0]	26.7 [25.7, 27.7]	26.5 [25.5, 27.5]	26.3 [25.4, 27.4]
HIP	−4.2% [−7.2, −1.1]	–	−3.7% [−7.0, −0.4]	−4.9% [−8.8, −0.9]	−3.6% [−6.4, −0.7]	–	−3.0% [−6.2, 0.2]	−4.5% [−8.4, −0.5]
GHQ-28	–	−5.1% [−11.0, 1.2]	−2.6% [−9.0, 4.1]	−3.2% [−9.5, 3.5]	–	−5.3% [−10.9, 0.5]	−3.1% [−9.3, 3.4]	−3.8% [−10.1, 3.0]
HIP × GHQ-28	–	–	–	3.3% [−3.1, 10.0]	–	–	–	4.0% [−2.3, 10.6]
Residual variation	20.6% [18.2, 23.4]	20.9% [18.4, 23.9]	20.6 [18.1, 23.5]	20.6 [18.1, 23.4]	21.1% [18.6, 24.0]	21.3% [18.8, 24.2]	21.0 [18.6, 23.8]	21.0 [18.5, 23.9]
Bayesian *R* ^2^	14.6 [7.1, 22.9]	12.3 [5.5, 20.3]	15.3 [7.6, 23.7]	16.0 [8.3, 24.3]	4.1 [0.2, 10.7]	2.4 [0.0, 7.5]	5.1 [0.5, 12.0]	6.4 [1.2, 13.8]
Model weights	10.2	0.9	4.3	3.7	31.3	10.2	19.5	19.9

HIP, HPV Impact Profile; GHQ-28, General Health Questionnaire-28; FSFI, Female Sexual Function Index; ZHIP, *Z*-score transformed HIP.

a. Estimates are reported as mean [95% credible interval].

b. FSFI ∼ 1 + ZHIP + baseline adjustment. (The ‘~’ acts as a formula separator in each statistical model, meaning ‘is modeled by’ or ‘is a function of’.)

c. FSFI ∼ 1 + GHQ-28 + baseline adjustment.

d. FSFI ∼ 1 + ZHIP + GHQ-28 + baseline adjustment.

e. FSFI ∼ 1 + ZHIP + GHQ-28 + baseline adjustment.

f. FSFI ∼ 1 + ZHIP. This model was the final selected model.

g. FSFI ∼ 1 + GHQ-28.

h. FSFI ∼ 1 + ZHIP + GHQ-28.

i. FSFI ∼ 1 + ZHIP + GHQ-28.

## Discussion

The purpose of the present study was to examine the moderating effect of general psychological health on the relationship between HPV-related psychosocial burden and QoL, as well as the relationship between HPV-related psychosocial burden and sexual function.

The negative impact of HPV infection on QoL stems from increased levels of anxiety and distress.^11–14^ Therefore, for the purposes of the present study, the LSI was selected as it is broader than the usual health-related QoL questionnaires and could thus contribute to a more accurate estimation of QoL. In addition, decreased QoL may have both HPV-related and non-HPV-related sources; thus, to assess the unique effect of HPV-related psychosocial burden on QoL, it was important to also account for the effects of more generic psychological well-being on QoL.

Our results suggested that both general psychological health and HPV-related psychosocial burden were independent predictors of QoL, although general psychological health alone was a stronger predictor of life satisfaction than HPV-related psychosocial burden alone (∼20% *v*. ∼9% of explained QoL variability) (Table 3, model 6 versus model 5). However, there was still a substantial unique effect of HPV-related psychosocial burden on QoL even after we had accounted for differences in general psychological health (model 7). For two random participants with the same general psychological health, higher HPV-related psychosocial burden was associated with ∼3% lower life satisfaction on average; thus, HPV-related impact on QoL was substantial even after accounting for the independent effect of general psychological health. This was further supported by the small increase in explained QoL variance (Table 3, model 3 versus model 2). Further, the mean difference in QoL between individuals with low and high general psychological health did not depend on the level of HPV-related psychosocial burden; that is, there was no further reduction in QoL associated with higher HPV-related psychosocial burden in women with no distress compared with women with distress (Table 3, model 8).

The LSI is affected by various physical, psychological and social factors that are considered to be important in HPV–infected women and can be used to evaluate QoL. Although HPV-related psychosocial burden is disease-specific, further reductions in QoL may result from other sources. In this regard, the inclusion of general psychological health was not directly specific to the HPV condition. GHQ-28 is used to detect possible psychological disorders and identifies two main concerns (inability to carry out normal functions and appearance of new and distressing phenomena).^26^ Previous studies have used both HPV-related psychosocial burden^12,27–32^ and various QoL tools^11–14^ to assess the impact of HPV infection. However, in those studies, the underlying constructs of the measurement tools were considered as outcomes and not as predictors. To our knowledge, the present study is the first to examine the strength of these tools as predictors of QoL. Higher HPV-related psychosocial burden was associated with ∼3% lower QoL on average, regardless of general psychological health; similarly, participants with worse general psychological health had ∼15% lower QoL on average regardless of HPV-related psychosocial burden. The fact that both tools detected significant effects implies that they should both be used in the clinical setting and that the underlying constructs potentially require different interventions to improve QoL in HPV-positive women.

On the contrary, HPV-related psychosocial burden, but not general psychological health, contributed as an independent predictor of female sexual function; however, the amount of explained variance was small. Decreased sexual function has been well established following HPV infection.^33,34^ In addition, a recent systematic review reported that 13 of the 15 included studies verified sexual dysfunction.^35^ The most frequent concerns reported by HPV-infected patients regarding sexual function included desire, arousal, genital response, orgasmic experience and satisfaction from orgasm.^36,37^ A verified HPV infection may result in loss of sexual desire or transformation of sexuality into an unpleasant experience;^38^ in fact, a negative impact on sexual function was reported in ∼87% of the analysed studies.^35^ Sexual dysfunction arises primarily from feelings of shame and loss of femininity that may also interfere with the relationship with the partner.^4,5^ In addition, the oncogenic potential of HPV infection is associated with increased anxiety and/or fear.^6,7^ Furthermore, Ferenidou et al^39^ reported, as well as impaired sexual desire, pain during intercourse and fear of transmission to the partner, which was exacerbated in patients with genital warts.^32^ These underlying effects on sexual function are probably too specific to have an impact on general psychological health; on the contrary, the seven domains of the HIP tool directly address these disease-specific issues.

Leite et al^29^ previously reported a positive correlation (∼0.34) between HPV-related psychosocial burden and sexual function. These researchers used the Index of Sexual Satisfaction, which consists of 25 items and assesses the degree of sexual dissatisfaction, with higher scores indicating lower levels of sexual dissatisfaction. Notably, the reported correlation had a small magnitude and was equivalent to a coefficient of determination (*R* ^2^) of 0.116, indicating that the amount of explained variance in sexual dissatisfaction was ∼11.6%. This correlation was higher than our reported values, but HPV-related psychosocial burden only accounted for ∼4–12% of the explained variance in sexual function. Therefore, there were contributing factors other than HPV-related psychosocial burden that would probably have needed additional tools to be detected. Comparing the impact of HPV-related psychosocial burden and general psychological health on QoL and sexual function, our best model accounted for approximately six times more variance for the former than the latter (model 7 (Table 3) versus model 5 (Table 4)). The stronger contribution of our predictors to QoL than sexual function may have been linked to fear of cervical cancer associated with HPV infection,^11^ worries about long-term health outcomes, concerns about relationships or stigma, and feelings of shame or guilt that arose immediately after diagnosis. Such reactions affect emotional well-being, which is a core component of QoL, but not necessarily of sexual function, at least not directly or immediately. Therefore, it is plausible that QoL is affected in women during the initial period following an HPV-positive diagnosis, whereas sexual function remains relatively unaffected or is not sufficiently impaired to produce a strong statistical effect. In addition, the wider spectrum of QoL probably incorporated variance associated with the more specific domain of sexual function.

Older women have reported greater sexual dissatisfaction 6 months after HPV diagnosis,^40^ which has led some authors to suggest that younger individuals may cope better with their intimate relationships and partners.^29^ In addition, specific HPV genotypes are associated with increased levels of anxiety, which adversely affect QoL.^33^ However, contrary to the results of these univariate analyses, we found that baseline demographic and clinical covariates did not enhance the predictive performance of HPV-related psychosocial burden and general psychological health; they may in fact have introduced some noise or redundancy. This does not imply that adjustment is not useful, especially as the effects of sociodemographic characteristics may vary according to cultural, social and economic traits of the population under investigation.^35^

Potential limitations of the present study should be noted. The cross-sectional design of the present study did not allow effects to be viewed as causal. This was a single-centre study; therefore, the sample size could be considered small, which may have caused certain strata to be underrepresented. The sample was recruited from a university gynaecology clinic; thus, it may have included a higher proportion of participants with active HPV compared with a random population sample. This could represent possible selection bias, as our sample may not accurately represent the prevalence of HPV in the Greek population. Further studies are required to elucidate the complex interplay between HPV-related psychosocial burden, sexual function and QoL. Follow-up studies in particular may reveal causal paths that affect sexual function and QoL. A potential limitation associated with the small amount of explained variance for sexual function was the lack of tools estimating the quality of the relationship between participants and their partners, as well as their satisfaction from the relationship. Future studies should consider using such tools to identify new predictors of sexual dissatisfaction in HPV-infected women. Despite these limitations, the findings highlight the distinct roles of psychological factors in HPV-related outcomes. General psychological health emerged as a stronger predictor of QoL in the initial period after diagnosis, beyond HPV-related psychosocial burden, underscoring the importance of early identification and management of mental health concerns to enhance counselling and psychological empowerment. In contrast, HPV-related psychosocial burden – but not general psychological health – significantly predicted sexual function, although a substantial proportion of the variance remained unexplained.

## Data Availability

The data supporting the findings of this study are available upon request by contacting the authors.
